# Effects of Summer Transhumance of Dairy Cows to Alpine Pastures on Body Condition, Milk Yield and Composition, and Cheese Making Efficiency

**DOI:** 10.3390/ani9040192

**Published:** 2019-04-24

**Authors:** Sudeb Saha, Nicolò Amalfitano, Enrico Sturaro, Stefano Schiavon, Franco Tagliapietra, Giovanni Bittante, Ilaria Carafa, Elena Franciosi, Luigi Gallo

**Affiliations:** 1 Department of Agronomy, Food, Natural resources, Animals and Environment (DAFNAE), University of Padova, Viale dell’Università 16, 35020 Legnaro (PD), Italy; sudeb.saha@studenti.unipd.it (S.S.); nicolo.amalfitano@studenti.unipd.it (N.A.); enrico.sturaro@unipd.it (E.S.); stefano.schiavon@unipd.it (S.S.); franco.tagliapietra@unipd.it (F.T.); bittante@unipd.it (G.B.); 2 Research and Innovation Centre, Edmund Mach Foundation, San Michele all’Adige, Via E. Mach, 1, 38010 San Michele All’adige (TN), Italy; ilaria.carafa@fmach.it (I.C.); elena.franciosi@fmach.it (E.F.)

**Keywords:** dairy cow, summer transhumance, alpine pasture, milk yield, milk coagulation properties, cheese yield

## Abstract

**Simple Summary:**

Summer transhumance of dairy cows is a seasonal pastoral system practiced in many European countries from ancient times. This practice provides additional forage supply for mountain dairy farms and plays a role in the preservation of landscape, biodiversity, and natural habitats and conservation of local traditional dairy products, but it may affect cows’ physiological and nutritional status. This study aimed to investigate the effects of transhumance of Brown Swiss cows to summer pastures on the yield, composition, and coagulation properties of milk, and on cheese yield. For this study, twelve multiparous cows from a mountain lowland permanent farm were divided into two groups of six cows: One group stayed at the permanent farm while the other moved to the alpine pasture (1860 m above sea level). Cows at the alpine pasture had reduced milk yield and body condition, and greater fat and lower protein contents in milk compared to cows at the permanent farm. Conversely, neither milk coagulation properties nor cheese yield were affected by summer transhumance. In conclusion, summer transhumance did not affect the cheese making efficiency of milk compared to permanent farm, but the negative effect on milk yield depressed daily cheese yield, which was 2 kg/d lower in cows moved to Alpine pasture.

**Abstract:**

Summer transhumance to alpine pastures (ALP) is widespread in dairy systems of alpine regions. This study aimed to investigate the effects of transhumance of Brown Swiss cows to ALP on the yield, composition, and coagulation properties of milk (MCP), and on cheese yield (CY). The study involved 12 multiparous cows kept at a mountain lowland permanent farm (PF), which were divided into two equal groups: One remained at the PF, the other was moved to the ALP (1860 m above sea level) from July to September. Every month (June to October), daily milk yield (MY) and body condition score (BCS) were recorded, and individual milk samples (*n* = 60, 2000 mL each) were collected to assess milk composition, MCP, and CY. Compared with PF, ALP cows had a reduced MY and BCS, which was maintained on return to the PF, greater fat and lower protein contents of milk. Neither MCP nor CY were affected by summer transhumance. In conclusion, summer transhumance did not affect the cheese making efficiency of milk but depressed MY and consequently daily cheese yield, which was nearly 2 kg/d lower for the ALP than the PF cows and was only partially recovered after returning to the PF in autumn.

## 1. Introduction

The transhumance of dairy cattle to temporary farms on mountain pastures during the summer months is an important, long-standing, traditional practice in the alpine regions of several European countries [1,2,3]. The practice has economic advantages for the alpine dairy sector, as it provides additional forage supply and may increase the added value of the milk obtained, destined mostly for the production of high-value local cheeses [4,5]. Moreover, transhumance and the management of summer pasture farms provide society and citizens with several positive services [6], by helping to preserve biodiversity and natural habitats [7,8], by contributing to tourism and recreational activities that help to preserve the traditional alpine landscape, cultural heritage, and established customs [9,10,11], and by helping to protect natural resources and the environment from natural hazards [3]. Lastly, dairy products from alpine pastures are presumed to be healthier, due to their favorable fatty acid profile [12,13], and are positively perceived by consumers for attributes related to taste, health, wholesomeness, and animal welfare [9,14].

When dairy cows are transhumed to summer alpine pastures, they undergo a number of changes compared with those that remain on permanent lowland farms, including different quality and availability of feedstuffs, regrouping of animals, adaptation to pastures, altitude-related hypoxia, and harsh climatic conditions [15,16]. Changes intrinsic to the alpine conditions may affect the cows’ nutritional status and metabolism, which in turn may influence milk yield, composition, and processing qualities. Several studies have evaluated the performance of cows and the characteristics of milk produced during summer transhumance, and a few of these have evaluated the animals and their milk before, during, and after summer transhumance [16]. However, studies comparing the cows kept on the lowland farms with those temporarily moved to highland pasture during the summer are scarce.

Based on these premises, this study aimed to investigate the response of Brown Swiss dairy cows to summer transhumance to alpine pastures in terms of the effects on body reserves, milk yield, composition, and coagulation properties, and individual cheese yield before, during and after transhumance compared with a lowland control group over the same period.

## 2. Materials and Methods

### 2.1. Farms, Animals, and Sample Collection

The trial was carried out from June to October with a dairy herd kept on a lowland permanent farm (PF; Malè, Italy, 737 m above sea level), and cows transhumed to a temporary summer highland farm (ALP; Malga Juribello, Italy, 1860 m above sea level), both located in the Trento province in the Northeastern Alps.

The PF herd comprised 92 lactating Brown Swiss that were loose-housed, milked in a milking parlor, and fed meadow and alfalfa hay and compound feeds. Every year around the end of June, part of the herd is moved from the PF to the ALP, where the cows are free to graze day and night on a typical *Nardetum alpigenum* association pasture, which has replaced the native woodland and shrub land [17]. The cows are moved around different areas of the pasture according to grass availability without a rigid rotation plan, and each is also given a compound feed supplement in the milking parlor.

According to these practices, in the current experiment, 12 mid-lactation multiparous cows were selected at the beginning of the trial (June) and allotted on the basis of their parity number and days in milk (DIM) to two treatments, one of 6 control cows, which remained on the PF throughout the trial, the other of 6 cows, which were transhumed to the ALP at the beginning of July, and back to the PF at the beginning of October. The cows in both treatments had similar (*p* > 0.05, based on t-test) parity numbers (2.5 and 2.8, respectively) and days in milk (DIM, 143 and 120, respectively).

The cows kept on PF were fed meadow and alfalfa hay (8 to 10 and 2 to 3 kg/d per head, respectively) and a commercial mixture of cereals, soybean meal, linseed, and maize germ meal (8.0 ± 2.0 kg/d, according to milk yield). Net energy and crude protein contents of the commercial mixture fed in the PF were 7.2 MJ and 150 g per kg as fed, respectively. In the ALP, the cows were kept at pasture, and received a commercial mixture of corn, wheat barn, soybean meal, sugarcane molasses, minerals, and vitamins as supplement (5.0 ± 1.5 kg/d, according to milk yield) during the milking. Net energy and crude protein contents of the commercial mixture fed in the ALP were 7.1 MJ and 140 g per kg as fed, respectively.

The following data were recorded and samples collected from all cows each month from June to October, during which period cows of one treatment remained continuously on the PF, the others on the PF in June and October and on the ALP from July to September:

- Individual daily milk yield (MY, kg/d);
- Individual milk samples (2000 mL per cow) collected during the evening milking and immediately refrigerated at 4 °C without preservative;
- Body condition score (BCS), assessed by a skilled operator on the same day as milk sample collection using the technique developed by Edmonson et al. (1989) [18], and expressed on a scale from 1 (thin) to 5 (fat) in increments of 0.25.

When cows were alternatively on the PF or on the ALP (from July to September), the monthly collection of milk samples was carried out in two subsequent days. All milk samples were transferred to the milk laboratory of the Department of Agronomy, Food, Natural Resources, Animals and Environment (DAFNAE) of the University of Padova and analyzed and processed the following morning within 20 h of collection.

### 2.2. Milk Quality Traits

Individual milk samples were analyzed for fat, protein, and lactose content with a Milkoscan FT2 infrared analyzer (Foss Electric A/S, Hillerød, Denmark) calibrated according to reference methods: ISO 8968-2/IDF 20-2 for protein [19], ISO 1211/IDF for fat [20], and ISO 26462/IDF 214 [21] for lactose. Somatic cell count (SCC) was obtained with a Fossomatic Minor FC counter (Foss Electric A/S) and log-transformed to somatic cell score (SCS) as proposed by Ali and Shook (1980) [22].

### 2.3. Milk Coagulation and Curd Firmness Properties

Curd firmness was measured on 120 milk samples (12 cows × 5 monthly samples × 2 replicates) every 15 seconds for 45 min (180 measures from each milk sample, in duplicate) using a lactodynamograph (Formagraph; Foss Electric A/S, Hillerød, Denmark) and according to the procedure described in detail by Stocco et al. [23]. In brief, a pendulum calibration was carried out before each session of the trial; 10 mL of milk was heated to 35 °C, then mixed with 200 μL of rennet solution (Hansen Standard 215 with 80 ± 5% chymosin and 20 ± 5% pepsin; Pacovis Amrein AG, Bern, Switzerland) freshly diluted to 1.2% (wt/vol) in distilled water.

We considered the traditional single point parameters of milk coagulation properties (MCP) reported by McMahon and Brown [24], namely, rennet coagulation time (RCT, min), defined as the time from rennet addition to milk gelation; curd-firming time (k_20_, min), defined as the time from gelation to a firmness of 20 mm within 45 min of enzyme addition; and curd firmness 30 and 45 min after rennet addition (a_30_ and a_45_, respectively, mm). In addition, we modeled all the curd firmness observations from each milk sample and estimated the individual curd-firming and syneresis equation parameters [25], namely, RCT_eq_ estimated from the individual curd-firming equations, the curd-firming instant rate constant (k_CF_), the curd syneresis instant rate constant (k_SR_), maximum curd firmness value (CF_max_), and time at CF_max_ (t_max_).

### 2.4. Cheese-Making Procedure and Traits

To assess cheese-making properties, 60 individual 1500 mL samples (12 cows × 5 monthly samples) were processed according to the model cheese-making procedure described in detail by Stocco et al. [26]. Briefly, each milk sample was poured into a stainless steel laboratory-vat, placed in a water bath (5 vats in each of 3 water-baths), heated to 35 °C (30 min), after which 8 mL of the same type of rennet used for the MCP analysis diluted to 4.29% in distilled water was added. After coagulation, the curd was cut, then drained for 30 min, and the resulting whey was measured for chemical composition using a Milkoscan FT2 infrared analyzer (Foss Electric A/S, Hillerød, Denmark). The curd was pressed for 30 min at 250 kPa by a cheese-pressing machine, turning every 10 min, then soaked in a brine solution (20% NaCl) for 30 min. After brining, the cheese wheels were weighed and pH measured with a Crison Basic 20 electrode (Crison Instruments SA, Barcelona, Spain). The following traits were computed from the weight and composition of the milk, whey, and curd: Three percentage cheese yield traits (%CY_CURD_, fresh cheese yield; %CY_SOLIDS_, total cheese solids yield; and %CY_WATER_, water retained in the curd); three percentage milk nutrient recoveries in the curd (REC_PROTEIN_, milk protein retained in the curd; REC_FAT_, milk fat retained in the curd; and REC_SOLIDS_, total solids retained in the curd); and daily cheese yield (dCY, kg/d), computed by multiplying MY by %CY_CURD_ and expressed as a daily measure.

### 2.5. Statistical Analysis

All the data were analyzed according to a linear mixed model (SAS 9.4, SAS Institute, Cary, NC, USA) that included the fixed effects of the Month × Treatment combination (8 levels: 4 months, July to October, and 2 treatments, PF and ALP), and the random effect of cow within Month × Treatment. The value measured in June was included as a linear covariate in the model for each trait to correct for possible initial differences among cows. Polynomial contrasts were estimated between the 5 least square means of month within PF to examine the response curve of each trait (linear, quadratic, and cubic components) during the 5 months in the cows kept on PF as a measure of the effect of season and lactation advancement. Contrasts between PF and ALP treatments were estimated separately within each month to test for the effect of transhumance to highland pasture during the summer months (July, August, and September) and the residual effect after returning to the PF (October).

## 3. Results

Table 1 shows the descriptive statistics and results of the mixed model for BCS, MY, composition, and MCP, and CY of cows kept on the PF or moved to the ALP.

The average MY was 25.7 kg/d, and fat and protein contents averaged 3.89 and 3.78%, respectively. The samples coagulated 22 min after rennet addition (RCT), and a curd firmness of 20 mm (k_20_) was obtained after 4.5 min. Average curd firmness at 30 min was 30 mm and increased to 42 mm at 45 min from rennet addition. On average, one kg milk yielded 160 g curd (66 g of solids, 94 g of retained water), producing 4.2 kg/d of curd. There were similar variations in MY and RCT, with coefficient of variation (CV) equal to 31 and 26%, respectively, whereas %CY_CURD_ had a much lower CV equal to 11%.

The combined Month × Treatment effect significantly affected most traits examined, as it combines the effects of advancing lactation within cow and changes in environmental conditions due to the advancement of the season with effects related to the different farming conditions. The covariate for the initial value (June) also reached statistical significance for several traits, allowing us to adjust the least squares means for possible differences among the cows at the beginning of the trial. Polynomial contrasts estimated between the least squares means of month for PF cows showed there were linear relationships between most traits and quadratic relationships between some traits with advancing season, but the cubic component never reached statistical significance.

**Figure 1:**
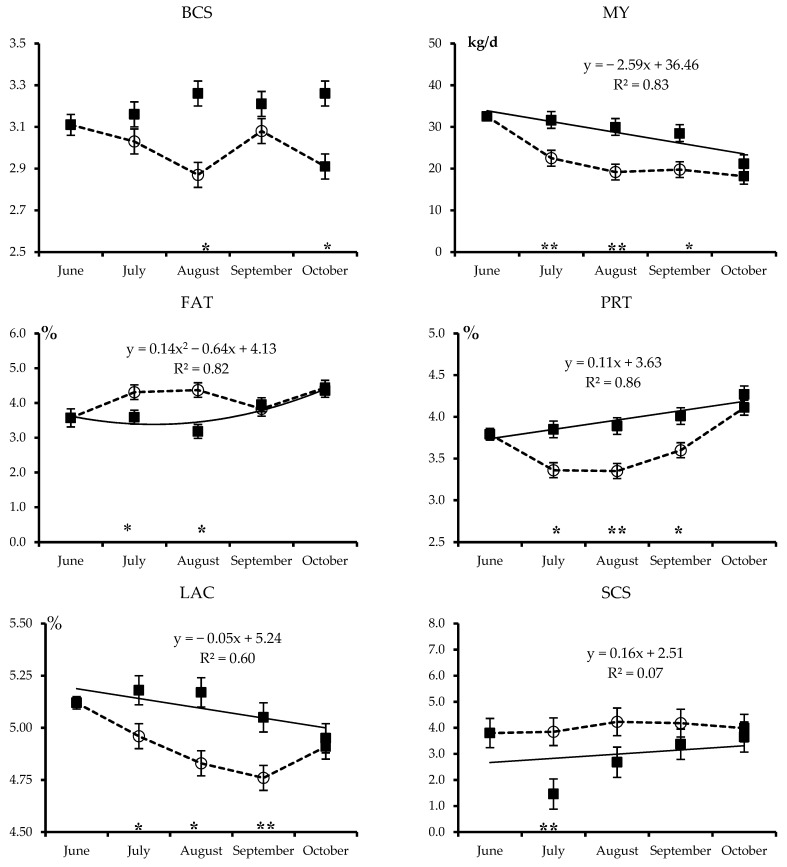
Least squares means and standard error of the combined Treatment × Month effect for body condition score (BCS) and milk traits (MY: Milk yield, FAT: Content of milk fat, PRT: Milk protein, LAC: Milk lactose, SCS: Somatic cell score) of Brown Swiss cows kept on the permanent farm (■ and continuous line) or moved to highland summer pasture from July to September (○ and dotted lines). Equations describe the trends of month within permanent farm, where statistically significant (R^2^ is shown). Asterisks refer to significant differences between cows moved to highland summer pasture and those kept on the permanent farm in a given month (* *p* < 0.05 or ** *p* < 0.01).

**Figure 2:**
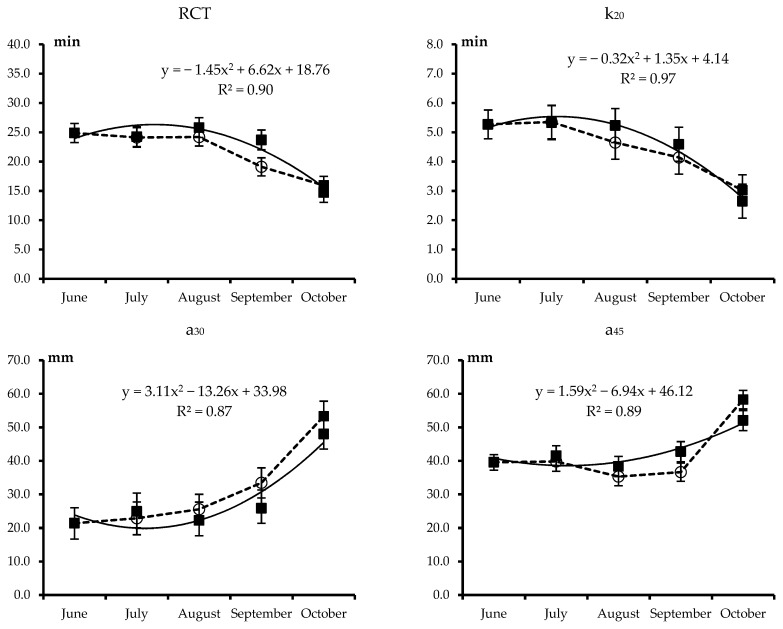
Least squares means and standard error of the combined Treatment × Month effect for milk coagulation traits (RCT: Rennet coagulation time, k_20_: Curd-firming rate as time to a curd firmness of 20 mm, a_30_ and a_45_, respectively: Curd firmness after 30 and 45 min from rennet addition) of Brown Swiss cows kept on the permanent farm (■ and continuous line) or moved to highland summer pasture from July to September (○ and dotted lines). Equations describe the trends of month within permanent farm (R^2^ is shown). Differences between cows moved to highland summer pasture and those kept on the permanent farm in a given month were never significant (*p* > 0.05).

**Figure 3:**
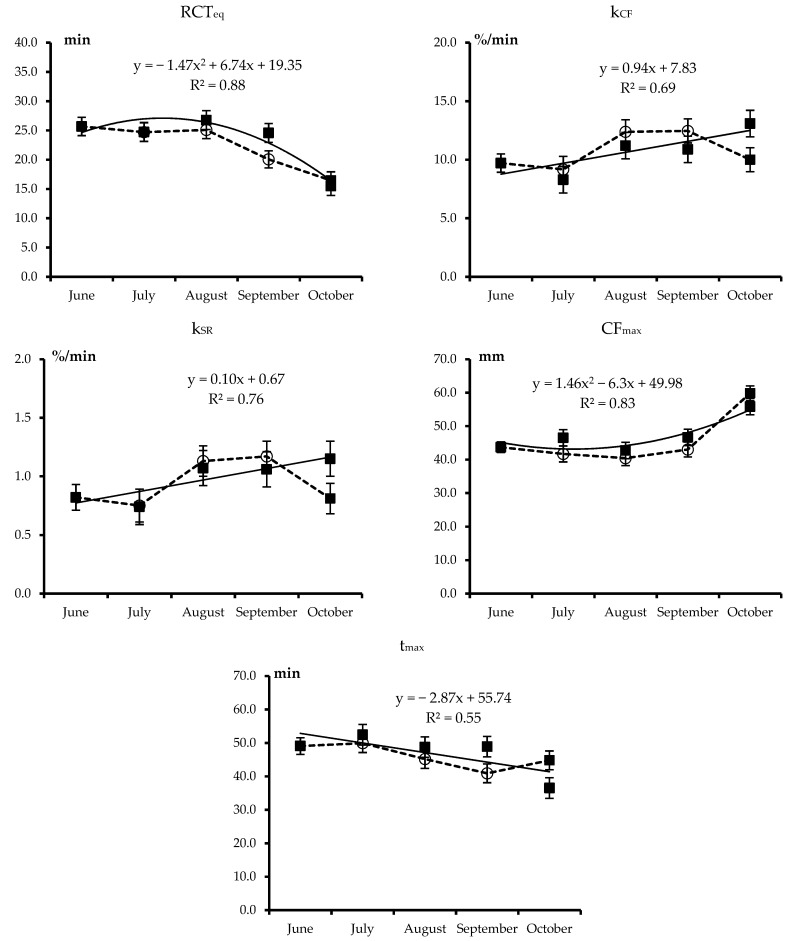
Least squares means and standard error of the combined Treatment × Month effect for curd firmness model parameters (RCT_eq_: Rennet coagulation time equation, k_CF_: Curd-firming instant rate constant, k_SR_: Syneresis instant rate constant, CF_max_: Maximum curd firmness, t_max_: Time to reach CF_max_) of Brown Swiss cows kept on permanent farm (■ and continuous line) or moved to highland summer pasture from July to September (○ and dotted lines). Equations describe the trends of month within permanent farm (R^2^ is shown). Differences between cows moved to highland summer pasture and those kept on the permanent farm in a given month were never significant (*p* > 0.05).

**Figure 4:**
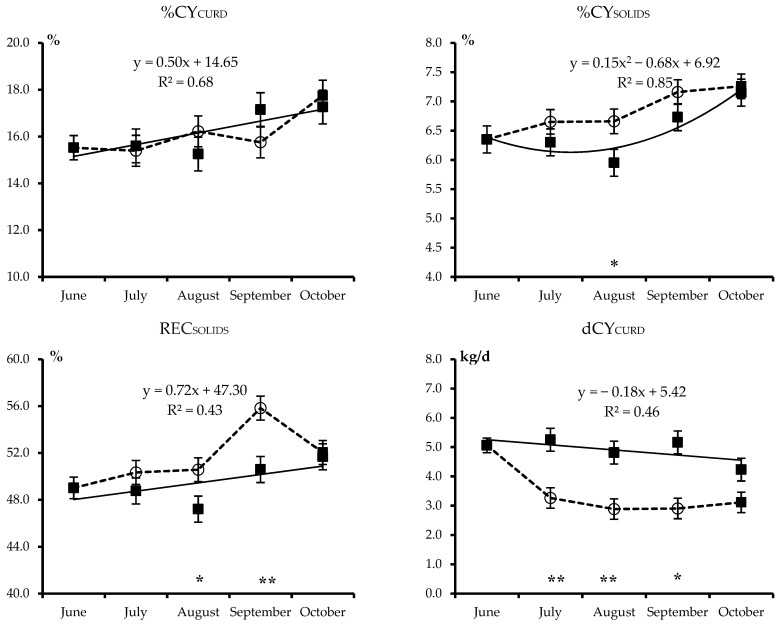
Least squares means and standard error of the combined Treatment × Month effect for fresh cheese yield (%CY_CURD_), total solids cheese yield (%CY_SOLIDS_), milk solids recovered in the curd (REC_SOLIDS_), and daily production of curd (dCY_CURD_) of Brown Swiss cows kept on permanent farm (■ and continuous line) or moved to highland summer pasture from July to September (○ and dotted lines). Equations describe the trends of month within permanent farm (R^2^ is shown). Asterisks refer to significant differences between cows moved to highland summer pasture and those kept on the permanent farm in a given month (* *p* < 0.05 or ** *p* < 0.01).

The least squares means of the combined effects of treatment and month for all analyzed traits are plotted in Figure 1, Figure 2, Figure 3 and Figure 4. Each figure also includes the linear or quadratic trends of the traits observed in PF cows, where statistically significant, and asterisks indicate significant differences between the ALP and PF cows in a given month.

The least squares means of BCS are shown in Figure 1. At the beginning of the trial, in June, BCS averaged 3.11 and remained essentially unchanged over the months for cows kept on the PF, reaching an average of 3.26 in October. However, the cows moved to the ALP lost body reserves, so their BCS was lower (*p* < 0.01) in August and in October after returning to the PF than that of the cows that remained on the PF.

Over the period June to October, the milk yield and lactose content of PF cows (Figure 1) decreased linearly, whereas protein and fat content increased linearly and quadratically, respectively, as expected due to the advancing of the stage of lactation. Transhumance notably reduced the MY of the ALP cows, which was 30–35% lower (*p* < 0.05) during the three months on the alpine pasture than that of the PF cows. The milk of the ALP cows also had lower protein and lactose contents than the milk of the PF cows during this period, but a greater fat content in the first (+20%) and second (+35%) months on the alpine pasture. The differences in milk yield and composition between the cows of the two treatments were less evident in October after the return of the transhumant cows to the PF.

The least squares means of the combined Treatment × Month effect for single-point MCP are reported in Figure 2. In general, the MCP of milk from PF cows tended to improve in a quadratic pattern from June to October, with advancing lactation stage. The RCT and k_20_ decreased (improved), especially in the last two months of the trial, while curd firmness traits (a_30_ and a_45_) increased in the same period. Transhumance did not affect single point MCP traits, which were nearly identical in the milk samples from both treatments for all months.

Least squares means of the combined Treatment × Month effect for the curd-firming model parameters are reported in Figure 3 and basically confirm the trends of the single-point MCP.

Namely, in PF cows, all CF_t_ parameters improved over the trial (and advancing stage of lactation), especially in September and October (quadratic trend) for RCT_eq_ and CF_max_. Transhumance of cows to summer alpine pasture did not affect the curd-firming model parameters, which mostly matched those of the milk from PF cows, so that cows of the two treatments had a common pattern of milk coagulation, curd firming, and syneresis.

Least squares means of the combined Treatment–Month effect for cheese yield and milk nutrient recovery in the curd are shown in Figure 4.

All traits reflecting the cheese-making efficiency of milk of PF cows, with the exception of REC_FAT_, also improved as the trial and lactation advanced. Curd yield (%CY_CURD_) and recovery of milk solids in the curd (REC_SOLIDS_) increased linearly from June to October, whereas the solids yield (%CY_SOLIDS_) increased in a quadratic trend, reaching maximum values in the samples taken in October. When moved to alpine pasture, the transhumant cows maintained the same %CY_CURD_ pattern as the PF cows, but %CY_SOLIDS_ was greater in August, and REC_SOLIDS_ greater in August and September (*p* < 0.05), returning to the same level as the PF cows after returning to the PF.

Daily curd production (dCY_CURD_, Figure 4) decreased linearly in the PF cows due to a combination of the strong linear decrease in MY and the slight linear increase in %CY_CURD_. There was a notable decrease in dCY_CURD_ (*p* < 0.01) over the entire summer alpine pasturing period, but on return to the PF, the difference between the two treatments did not reach statistical significance.

## 4. Discussion

### 4.1. Body Condition Score and Milk Yield and Composition

The progressing months of the trial reflect the effects of advancing stage of lactation, which passed from an average of 140 DIM at the beginning to 260 DIM at the end of the trial. The pattern of change in MY, milk composition, and SCC was generally consistent with expectations for cows progressing from mid- to late lactation, namely, a decrease in MY and lactose content, and an increase in fat and protein contents and SCC [27,28].

Transhumance to summer alpine pasture has notable effects on the cows’ physiological, social, feeding, and nutritional status, which may affect performance and milk quality traits [16]. It involves changing from indoor barns to outdoor rearing and from a feeding strategy of mostly constant rations to grazing on pastures, frequently characterized by low productivity, limited nutritional value, sward with a high fiber content, and seasonal variation [15,29]. In addition, the cows’ activity increases through walking long distances on steep grazing areas, which may increase their energy expenditure and limit their grass intake, with negative effects on nutritional status. Transhumance to alpine pasture has been frequently associated with a reduction in feed intake [15,30,31], which can reduce the energy available for metabolism. The resulting deficiency in nutrients and energy, and changes in the environment and management may explain the reduction in MY and the protein and lactose contents of the milk of the ALP cows compared with the PF cows throughout the entire summer pasturing period. The lower MY after transhumance is consistent with the findings of several studies, which report decreases in magnitudes ranging from 10 to over 40% [5,15,29,32]. Increased milk fat content associated with the transfer of lactating cows to summer alpine pasture has also been reported by Leiber et al. [15], Bergamaschi et al. [5], and Niero et al. [32], although Bugaud et al. [33] and Zendri et al. [29] did not find this to be the case. The increase in milk fat content has been attributed to greater body fat mobilization, which characterizes cows kept on alpine pastures compared with cows on permanent farms [15]. In the present study, we found different patterns of change in BCS in PF and ALP cows, the latter having lower scores both during alpine pasturing and after returning to the PF compared with the former. This is consistent with the findings of Leiber et al. [31,34], who reported greater body fat mobilization and increased blood plasma levels of β-hydroxybutyrate and non-esterified fatty acids in cows kept in alpine conditions.

Alpine pasturing of cows has been frequently reported as having a negative effect on milk protein contents [35,36,37], presumably due to hypoxia and a deficient nutrient supply [34], despite the increasing practice of supplementing the cows’ diet with concentrates [38]. A decrease in lactose content after transhumance to summer alpine pasture has also been reported by Romanzin et al. [37], Bergamaschi et al. [5], and Zendri et al. [29], although other studies found no change [15,32].

### 4.2. Milk Coagulation Properties, Curd Firming, and Syneresis

In this study, the MCP parameters were on average comparable to those reported by Cecchinato et al. [39] and Stocco et al. [23] for the milk of Brown Swiss cows in mid- and late lactation kept on mountain lowland farms. Rennet coagulation time, curd-firming time, and curd firmness of the milk from PF cows varied with month of trial (and days in milk) in a quadratic pattern: RCT and k_20_ remained stable until August, when the average DIM of cows approached 200 days, and decreased thereafter; whereas curd firmness after 30 and 45 min (a_30_ and a_45_, respectively) increased in the last month of trial. Bittante et al. [40] also reported a quadratic pattern of change in RCT, curd-firming time, and curd firmness with advancing stage of lactation: Milk coagulation properties decreased in the first half of lactation and progressively recovered thereafter, confirming the results of Malchiodi et al. [41]. Our results also agree with the findings of Stocco et al. [23], who found an improvement in k_20_ and a_30_ in the second half of lactation. The pattern of change in the curd firmness model parameters of milk from PF cows as the trial progressed and DIM increased was consistent with the results of Bittante et al. [40], confirming an improvement in milk coagulation ability in the last stage of lactation.

Alpine pasturing of dairy cows has been reported to impair the rennet coagulation time of the milk [34,42]. An increase in the RCT of milk from these cows compared with cows kept in lowland barns was reported by Niero et al. [32], who did not however observe differences in curd firmness between the treatments of cows. Leiber et al. [15] reported impaired RCT and k_20_ in milk from cows grazed at high altitude and attributed this decrease in rennet coagulation properties mainly to the lower protein content of their milk. It is worth noting that studies on summer transhumance frequently compare lowland and highland conditions using data from the same group of cows before and during summer pasture, an experimental design that confounds the effects of changing the environment/feeding with those of advancing lactation/pregnancy. As the cows of the two treatments in our study were from the same herd, were kept in the same conditions until June, and then again in October, and had similar parities and DIM, the only difference between them was that one spent the period July–September on the ALP while the other remained on the PF. Summer pasturing of the cows, despite reducing the milk protein content, did not alter the milk coagulation properties and curd-firming process, so that the patterns of these during the entire period on the alpine pasture and after returning to the PF in the autumn were the same as those of the PF cows. Our results are partly consistent with the findings of Zendri et al. [16], who reported that moving cows to summer pasture did not affect coagulation time and only slightly influenced the curd-firming process. Bovolenta et al. [4] suggest that feeding concentrates to cows on mountain pasture would improve milk coagulation properties. It is therefore possible that inconsistent results concerning the effects of summer alpine pasturing on MCP may be partly due to differences in concentrate supplementation, which may mitigate the potentially detrimental effects of summer alpine pasturing on MCP.

### 4.3. Cheese Yield and Nutrient Recovery in Curd

The average percentage fresh cheese yield (%CY_CURD_) found in this study is comparable to that reported by Cipolat-Gotet et al. [43] using the same model-cheese manufacturing process and milk from Brown Swiss cows in mid- and late lactation reared in mountain lowland farms. All %CY traits of milk from PF cows increased with the progressing month of the trial and DIM, with patterns of variation consistent with those reported by Cipolat-Gotet et al. [43] and Stocco et al. [26], who observed a progressive increase in %CY_CURD_, %CY_SOLIDS_ and REC_SOLIDS_ from the second month to the end of lactation. This pattern is consistent with the observed improvement in milk nutrients contents.

The authors are unaware of any study comparing the cheese yield of milk obtained during summer pasturing with that of milk obtained in the lowlands. The average %CY_CURD_ values found in this study using the individual model cheese-making procedure were slightly higher than those obtained by Bergamaschi et al. [5] in cheeses made from milk from the same ALP as this study but using an artisanal cheese-making procedure. The values for milk nutrient recovery in the curd found here are also in agreement with those reported by Bergamaschi et al. [5].

In the current study, the summer pasturing of cows did not alter the %CY_CURD_, and ALP and PF cows had similar fresh cheese yields during the entire period of alpine pasturing, although the cows at pasture had somewhat greater %CY_SOLIDS_ and REC_SOLIDS_ than those kept on the permanent farm. The differences between the treatments remained more or less the same after the cows returned from the alpine pasture to the PF in autumn. In a survey of 15 temporary summer farms, Zendri et al. [16] predicted cheese yield traits on the basis of Fourier-transform infrared spectrometry of milk samples. They reported a negative effect of the transhumance on %CY_CURD_ in the first month at pasture, but thereafter a progressive increase in fresh cheese yield, coinciding with the advancing of DIM and the cows adapting to the conditions after the initial stress. However, without a lowland control group, the study’s experimental design confounds the effects of DIM with the effects of transhumance.

Daily fresh cheese yield decreased linearly in PF cows with advancing DIM, a trend that was expected and consistent with the findings of Cipolat-Gotet et al. [43] on lowland farms. The differences between animals of the two treatments in dCY_CURD_ reflect differences in MY and %CY_CURD_ between the two groups: During the months at pasture, ALP cows produced nearly 2 kg/d less fresh cheese than PF cows, a gap that narrowed only partially after their return to the permanent farm in autumn.

## 5. Conclusions

This study presents new information on the effects that summer transhumance of dairy cows to alpine pastures exerts on the yield, composition, and cheese-making properties of milk, by comparing the performance of two homogeneous groups of cows from the same permanent farm before, during, and after the transfer of one group to summer highland pastures.

The results show that transhumant cows had lower body condition scores, and reduced milk yield and milk protein content, although milk traits were partially recovered after their return to the lowland permanent farm. From this, we can confirm that transhumance to alpine pastures may affect productive functions and body fat reserves, even when cows are given concentrate supplements. On the other hand, the cheese-making attributes of milk were not affected by summer transhumance: Milk coagulation properties, curd-firming parameters, and cheese yield were nearly identical for cows transferred to alpine pasture and those that remained on the lowland permanent farm. A consequence of the adverse impact of alpine pasturing on milk yield was the substantially lower daily cheese yield from transhumant cows than from cows remaining on the permanent farm, although this effect tended to narrow after the cows returned to the permanent farm. The production losses resulting from alpine grazing need to be compensated for by creative marketing of alpine products and adequate remuneration of the environmental, recreational, social, and cultural services provided by the transhumance of dairy cows to mountain pastures.

## Figures and Tables

**Table 1:** Descriptive statistics (mean ± standard deviation) and results from the mixed model for body condition score (BCS), milk traits, milk coagulation properties (MCP), curd-firming modeling parameters (CF_t_), percentage cheese yield (%CY), milk nutrients recovery in curd (REC), and daily yield of curd (dCY_CURD_) of cows kept on the permanent farm or moved to temporary summer pasture (Treatment) during the June to October interval (Month): F-values and significance (* *p* < 0.05; ** *p* < 0.01) for the combined Treatment × Month fixed effect, and for the covariate on the initial value of the trait (COV); percentage of variance explained by the random effect of cow on total variance (Cow); *P*-value of the linear (L), quadratic (Q) and cubic (C) trends of the trait from June to October within permanent farm (TREND).

Traits	Variables	Mean ± SD	Month × Treatment	COV	Cow	RMSE ^1^	*P*-Value of TREND		
							L	Q	C
BCS and milk traits	BCS	3.09 ± 0.21	5.83 **	25.88 **	2	0.14	0.39	0.70	0.39
	Milk yield, kg/d	25.71 ± 8.04	6.09 **	4.51 *	32	3.32	<0.0001	0.07	0.37
	Fat, %	3.89 ± 0.72	4.70 **	0.48	8	0.46	0.002	0.05	0.10
	Protein, %	3.78 ± 0.31	25.06 **	5.63 *	53	0.13	<0.0001	0.06	0.81
	Lactose, %	5.01 ± 0.21	5.57 **	6.53 *	57	0.10	0.0005	0.38	0.50
	SCS (somatic cell score)	3.49 ± 1.62	2.72 *	7.38 *	36	1.03	0.002	0.32	0.95
Single-point MCP ^2^	RCT, min	21.99 ± 5.80	8.54 **	13.95 **	21	3.34	<0.0001	0.002	0.64
	k_20_, min	4.53 ± 1.57	5.65 **	2.28	40	0.99	0.003	0.05	0.70
	a_30_, mm	30.14 ± 16.44	8.11 **	8.32 **	26	9.43	0.002	0.01	0.57
	a_45_, mm	42.43 ± 9.14	14.00 **	0.19	40	5.12	0.002	0.01	0.79
CF_t_ parameters ^3^	RCT_eq_, min	22.77 ± 5.70	9.47 **	14.47 **	20	3.21	<0.0001	0.001	0.68
	k_CF_, %/min	10.74 ± 3.14	3.12 *	15.09 **	25	2.19	0.004	0.72	0.20
	k_SR_, %/min	0.96 ± 0.38	2.50 *	10.35 **	30	0.27	0.03	0.35	0.42
	CF_max_, mm	46.29 ± 7.80	17.21 **	1.29	46	4.02	0.001	0.001	0.76
	t_max_, min	46.36 ± 8.94	3.90 **	14.22 **	23	6.00	0.001	0.12	0.19
%CY, %	Curd	16.06 ± 1.82	2.37 *	5.05 *	17	1.46	0.03	0.72	0.18
	Solids in curd	6.62 ± 0.70	4.80 **	0.54	24	0.44	0.001	0.06	0.11
	Water in curd	9.44 ± 1.42	2.40 *	1.13	33	1.08	0.11	0.74	0.25
REC, %	Fat	83.18 ± 4.35	0.69	1.43	19	0.64	0.23	0.82	0.84
	Protein	78.61 ± 1.67	2.98 *	40.11 **	54	0.41	0.37	0.19	0.08
	Solids	50.34 ± 3.39	6.88 **	0.67	32	2.04	0.01	0.16	0.09
Daily yield of curd	dCY, kg/d	4.16 ± 1.30	3.81 **	1.05	32	0.68	0.06	0.43	0.14

^1^ RMSE = root mean squared error. ^2^ Single-point MCP: RCT = rennet coagulation time; k_20_ = curd-firming rate as min to a curd firmness of 20 mm; a_30_ (a_45_) = curd firmness after 30 (45) min from rennet addition. ^3^ Curd firming: RCT_eq_ = rennet coagulation time estimated from the equation; CF_P_ = asymptotic potential curd firmness; k_CF_ = curd-firming instant rate constant; k_SR_ = syneresis instant rate constant; CF_max_ = maximum curd firmness attained within 45 min; t_max_ = time to reach CF_max_.
